# Analysis of deformation mechanism of rainfall-induced landslide in the Three Gorges Reservoir Area: Piansongshu landslide

**DOI:** 10.1038/s41598-024-60590-w

**Published:** 2024-05-01

**Authors:** Hui Wang, Jianhua Zou, Xinghua Wang, Peng Lv, Zefu Tan, Longfei Cheng, Qiang Wei, Binli Qin, Zhengchao Guo

**Affiliations:** 1 School of Civil Engineering, Chongqing Three Gorges University, Chongqing, 404020 China; 2 Sichuan Geological Environment Survey and Research Center, Chengdu, 610081 China; 3 Institute of Geographic Sciences and Natural Resources Research, Chinese Academy of Sciences, Beijing, 100101 China

**Keywords:** Accumulation landslides, Rainfall, Deformation mechanism, Stability evaluation, Civil engineering, Natural hazards

## Abstract

The Three Gorges Reservoir Area (TGRA) is characterized by unique geological features that increase its susceptibility to landslides. These slopes are especially prone to destabilization when influenced by external triggers like rainfall. This research focuses on the Piansongshu landslide within the TGRA, aiming at unraveling the complex internal deformation mechanisms of landslides triggered by rainfall and providing critical insights for their prevention and mitigation. The study begins with on-site geological surveys to meticulously examine the macroscopic signs and mechanisms of deformation. It then utilizes the GeoStudio numerical simulation software to assess the landslide's stability, focusing on the changes in internal seepage fields and stability under various rainfall scenarios. Results indicate that continuous rainfall leads to the formation of a temporary saturation zone on the slope, which gradually deepens. In regions with more pronounced deformation, the infiltration line at the leading edge of accumulation notably protrudes towards the surface. Notably, the stability coefficient of the secondary shear surface of the landslide fluctuates more significantly than that of the primary sliding surface. Higher rainfall intensity and longer duration are positively correlated with a more pronounced decrease in stability coefficients. The impact on stability also varies across different rainfall patterns. As rainfall infiltrates over time, the slope's safety factor gradually decreases. This reduction continues even post-rainfall, indicating a delayed restoration period before stability returns to a safe level. These results yield valuable data for forecasting and mitigating landslides.

## Introduction

Landslide disasters, a prevalent issue not only in China but globally, account for a significant proportion of geological disasters^1^. In southwestern China, regions such as Chongqing, Sichuan, and Guizhou are particularly prone to landslide disasters^2–4^. The formation and evolution of landslides in the Three Gorges Reservoir Area (TGRA) are influenced by various dynamic and internal factors. Among these are regional geological background, reservoir water level fluctuations and rainfall^5,6^, as well as slope structure^7^, sliding surface morphology^8^, wading degree and permeability characteristics^9^. Reasonable evaluation of landslide stability is crucial for the prevention and control of geological disasters. This evaluation process begins with the establishment of a geological model based on thorough geological analysis^10^. The complex reality is then distilled into a solvable mathematical model, facilitating the derivation of stable results through calculations^4^. A field geological survey is a crucial prerequisite for landslide stability calculation, while numerical simulation serves as a crucial method for stability assessment. This requires a comprehensive analysis of the factors influencing landslides and their formation mechanisms, all within the geological context^11^.

Landslides are the outcome of a complex interplay of various factors, each deeply rooted in the geological environment of the affected area. By combining on-site monitoring with extensive analysis, it's possible to unravel the diverse elements and mechanisms contributing to landslides. Fluctuations in water levels are known to negatively impact slope stability^12^. On a microscopic scale, clay minerals play a significant role in the development of landslides. The shear strength parameters of the soil in the sliding zone, which are crucial in controlling the stability and evolution of landslides, are subject to some uncertainties and thus warrant thorough characterization and consideration in stability analyses^13^. Moreover, precipitation is a critical factor driving landslide occurrences^14^. The key triggers for landslides are rainfall infiltration and the presence of weakly saturated zones at specific depths. Computed Tomography (CT) visualization technology was utilized to monitor and scan the failure process of the shear zone in real-time and to investigate the failure mechanism^7^. The stability of the landslide is evaluated by combining numerical models, and the accuracy of the modeling method is verified by field geological investigation^15^. The process replicates the landslide’s dynamic response to external forces and the movement and accumulation of rock masses^16^. A cost-effective and practical integrated early warning system has been developed for shallow landslides in a specific area^17^. The system takes into account the rainfall threshold and slope stability to accurately predict landslides. The stability of landslides under the coupling of multiple factors is studied by combining the late creep of the rock-soil mass^18^. Based on the GPS monitoring data of the landslide after water storage, the stability of the landslide is studied with the response of rainfall and reservoir water level^19,20^. To analyze the landslide in the context of the combined effects of water level fluctuations, rainfall-induced damage patterns, and deformation mechanisms, physical experiments were undertaken^21^. The relationship between critical hydrological state, rainfall intensity, and soil properties was established, and the variation of landslide damage surface depth was determined^10^.

As technology continues to progress, the utilization of data integration and analysis techniques has become increasingly popular in the field of landslide prediction and assessment^22^. This process involves merging applied mathematics with computational intelligence to classify or perform regression tasks on unknown data^23^. Machine learning methods help in landslide modeling and predicting the occurrence of landslides with robust data structures, and building algorithmic models without human intervention and prior assumptions^24^.

Using polygonal surfaces with higher reliability and accuracy to represent landslide boundaries and spatial shapes can significantly improve the accuracy of landslide hazard zone delineation^25^. Representing landslide boundaries as circles introduce errors in measurements and is less accurate than polygonal boundaries in most cases^26^.

Among the landslides that have occurred in the TGRA, accumulation landslides are both the most numerous and the highest proportion of occurrences. These landslides are prone to various degrees of deformation under the influence of external triggers such as fluctuations in water levels and rainfall. Destabilization occurs when the accumulated deformation reaches a critical threshold. Despite previous research efforts, there remains a need for further investigation into the deformation mechanisms of these layered landslides. Located in the hinterland of the TGRA, the Piansongshu landslide represents a typical example of a rainfall-triggered, stacked layer landslide in the region. Geological conditions primarily govern the deformation and destruction of this landslide, with rainfall serving as the principal trigger factor. Building upon geological analysis, this study employs a approach by integrating multi-field coupling evaluation of landslides with the transfer coefficient method. Through this methodology, the study delves into the internal deformation mechanisms and stability of landslides under varying rainfall conditions, thereby shedding light on the deformation mechanisms to rainfall-triggered landslides. This innovative study not only combines geological analysis with numerical simulation but also incorporates the transfer coefficient method, offering reference for the deformation mechanisms of rainfall-triggered landslides in the region.

## Review of the Piansongshu landslide

### Regional accumulation layer landslide distribution law

**Figure 1:**
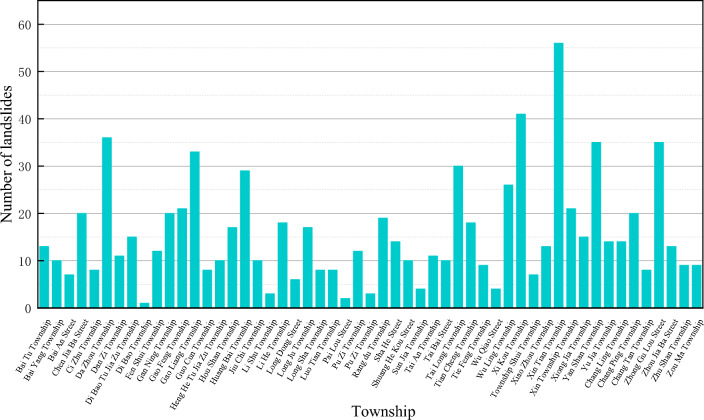
Distribution of Landslide Disasters Across Townships in Wanzhou District.

The pattern of regional accumulation landslides within the TGRA is predominantly dictated by the underlying regional geology. Specifically, in the Wanzhou district, a notable concentration of landslide accumulations can be observed along the riverbanks (Fig. 1). The prevalent landslide hazards in this area are soil landslides, which often manifest in distinctive skip and tongue-shaped formations across the landscape. These formations are intricately linked to the geological backdrop of the Wanzhou district, marked by its terraced landforms that promote the concentrated emergence of these layered landslides. Moreover, the interplay of reservoir water level fluctuations and rainfall significantly influences the propensity for these landslides to develop. When analyzed in profile, most landslides in the Wanzhou district demonstrate stepped and linear geometries. Their sliding planes generally correspond to the interface between the overlying soil layer and the underlying bedrock, with these events typically unfolding on inclines that range from gentle slopes of 10° to steep escarpments of 40°. The hydrogeology within these landslides is complex, comprising predominantly of pore water, fissure water, and seepage water. The landslides' planar shapes are sculpted by a mix of factors such as topography, the composition of geological strata, and the external forces exerted upon the slopes. The spatial distribution of landslides further delineates the edge constraints of these geological phenomena and the structural characteristics of the affected slopes. Among the catalysts for landslide calamities in the Wanzhou district, torrential rainfall stands as the foremost trigger, with seismic activities and anthropogenic interventions also contributing to their incidence.

### Landslide profile

**Figure 2:**
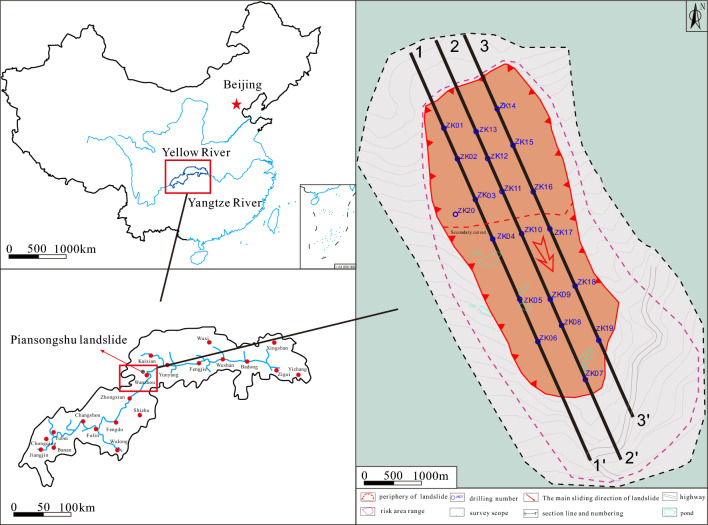
Plane form of the Piansongshu landslide.

**Figure 3:**
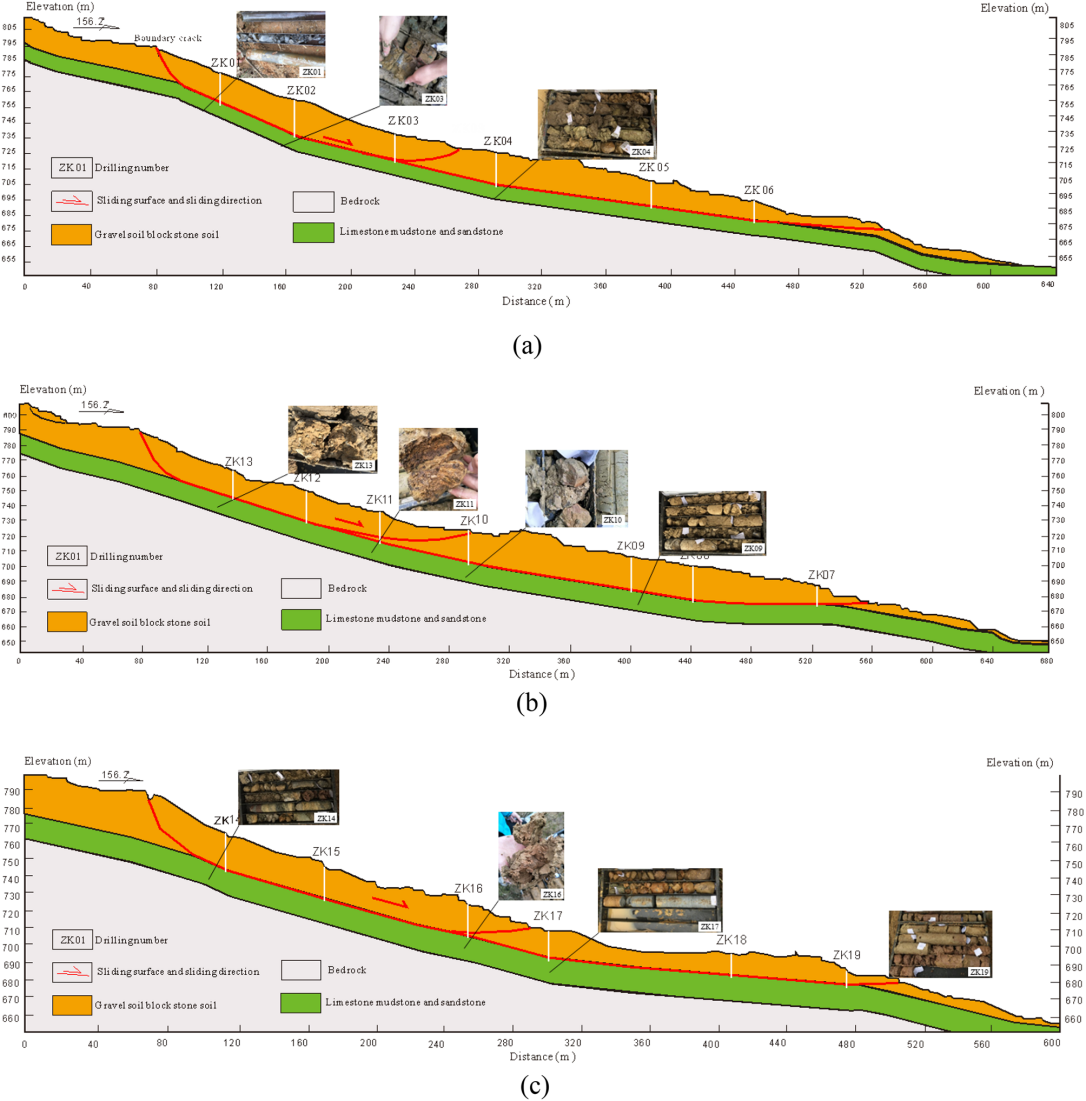
Longitudinal profile of the Piansongshu landslide.

The Piansongshu landslide is located in the slope region of Dabei Village, Gaoliang Town, Wanzhou District (Fig. 2). The landslide has a tongue-shaped plane form with a longitudinal length of 470 m and a transverse width of approximately 150–190 m. The distribution area covers around 7.57 × 10^4^ m^2^, with an average thickness of 13 m and a volume of approximately 98.4 × 10^4^ m^3^. The main slide direction of the landslide is around 156°, and it is classified as a medium-sized soil landslide. Based on the revealed morphology of the slip surface from drilling, the landslide exhibits a concave-shaped sliding surface, characterized by a steeper back and a gentler front. From the three longitudinal sections of the landslide (Fig. 3), the overall average slope of the landslide is approximately 25°, with the middle and rear parts averaging around 30°, while the average slope angle of the front part is approximately 15°. The section shape of the landslide shows stepped features with both steep and gentle slopes, as well as staggered platforms.

The Piansongshu landslide mainly slides along the contact surface of soil and bedrock. According to the drilling reveal, the surface of the landslip bed changes from steep to slow from back to front and has a concave form in the profile. The landslide is predominantly composed of gravel soil and block gravel soil, with gray-black chert and yellow–brown sandstone as the main types of limestone present. Silty clay is interlayered between the gravel and block materials. Drilling investigations indicate that the landslide is characterized as a soil slide occurring along the rock-soil contact surface, with sliding primarily taking place along soil cracks at the trailing edge. The sliding zone is mainly distributed at a certain thickness above the bedrock surface. The soil within the slide zone consists of silty clay containing gravel, with the gravel predominantly composed of limestone and sandstone. Internally, the soil within the slide zone exhibits wrinkle-like features. The slide bed is composed of bedrock, mainly limestone of the Middle Triassic Badong Formation (T_2_b), interspersed with dolomitic marl, purple-red mudstone, and similarly colored yellow–brown sandstone.

### Macroscopic deformation characteristics of landslide

**Figure 4:**
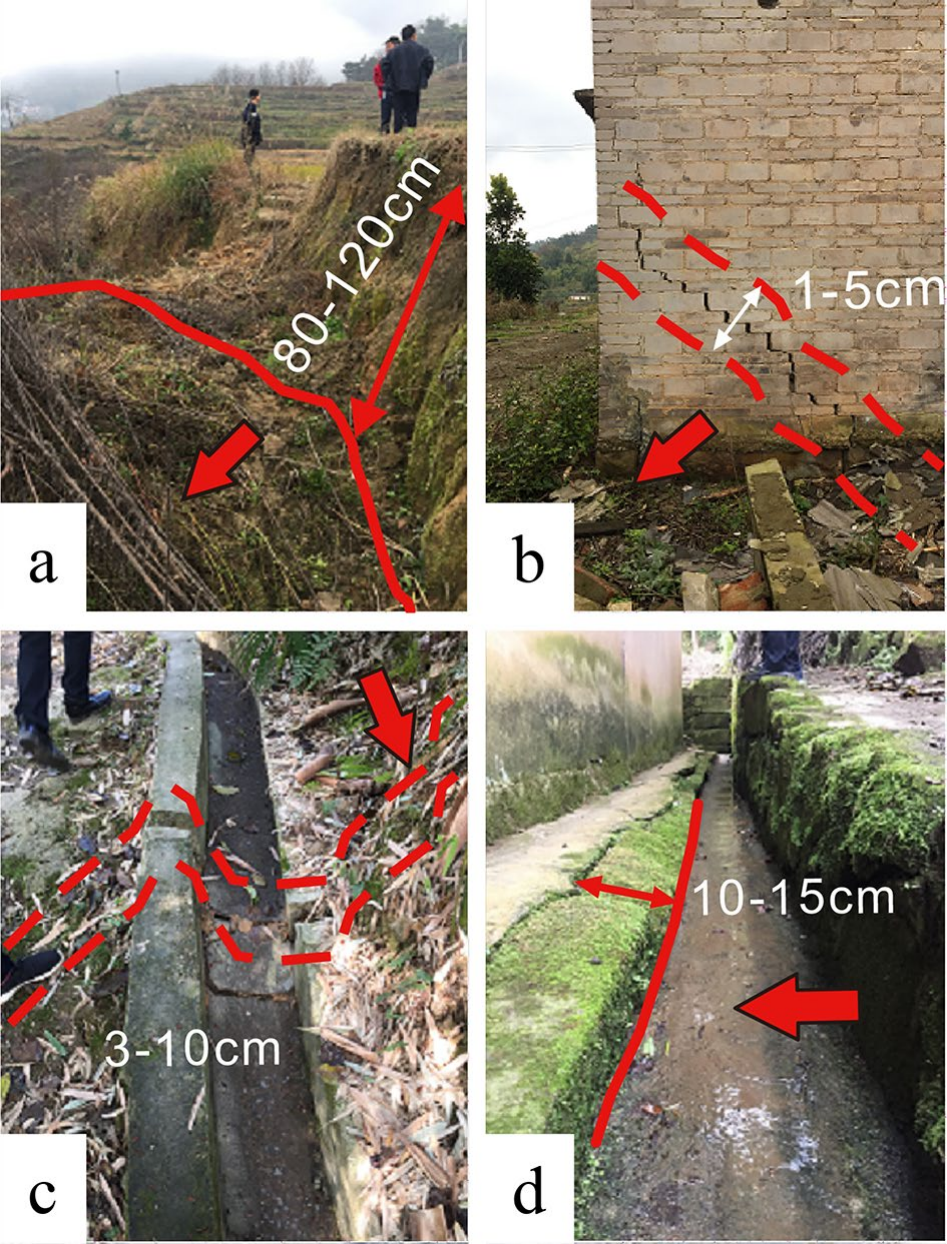
Deformation signs of the upper landslide area. (**a**) Cracks in the trailing edge of the landslide; (**b**) Lateral cracks in the middle and rear houses of landslide; (**c**) The right ditch crack in the middle of the landslide; (**d**) Gully swelling in the middle of landslide.

Based on field geological investigations, the secondary shear zones of landslides (Fig. 4) are mainly characterized by the separation and pulling back of the trailing edge, the appearance of additional cracks in nearby buildings, and increased deformation of pre-existing cracks. In the middle of the landslide, the front yard dam bulged and cracked, and the gutter extrusion gradually increased. The soil at the back edge of the landslide showed tension cracks, and the deformation of the cracks at the back edge appeared to be deflected (with the widest crack measuring 50 cm and a deflection distance of 120 cm) with the frequent occurrence of heavy rainfall, leading to intensified deformation for a short period and expanded range, which is influenced by the rainfall.

**Figure 5:**
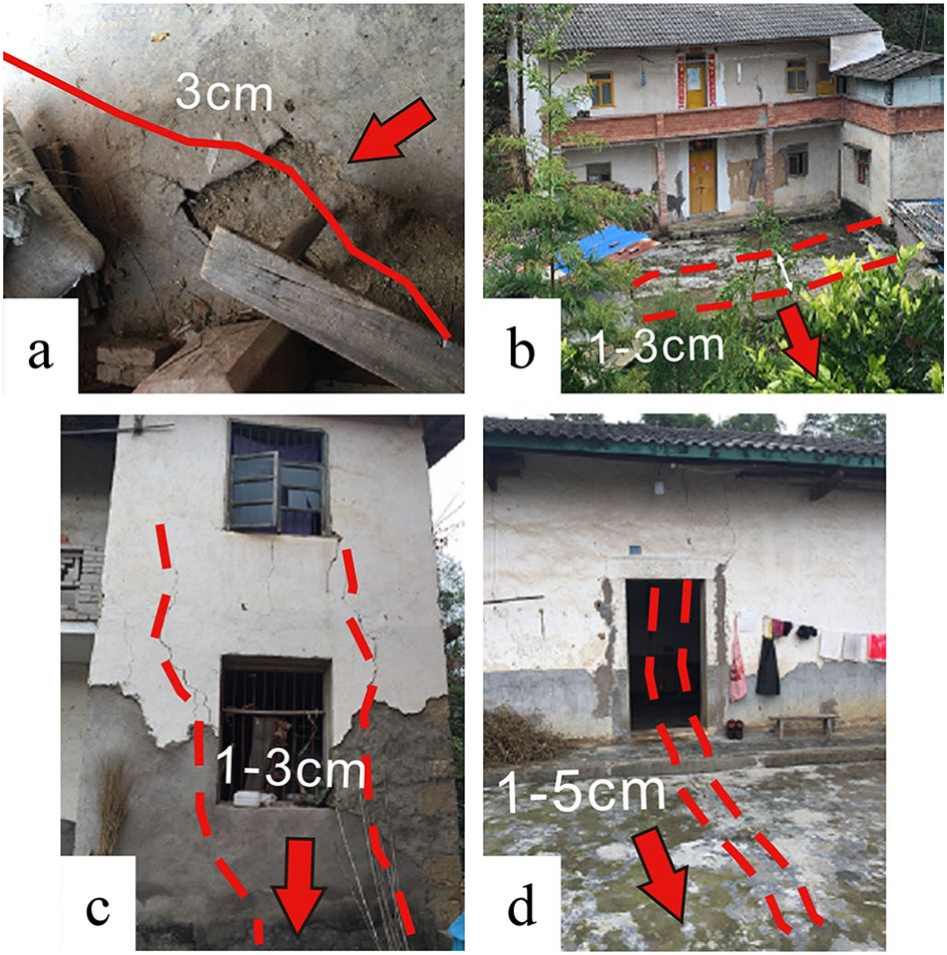
Deformation signs of the lower landslide area. (**a**) The ground on the left side of the front edge of the landslide uplifts; (**b**) Transverse cracks in the left house in the middle of the landslide; (**c**) Longitudinal cracks in the front house of the landslide; (**d**) Longitudinal cracks in the front house of the landslide.

The lower part of the landslide area (Fig. 5) mainly exhibits Dam bulging uplift of the front edge yard dam and cracking of the building. Field geological investigation reveals a significant increase in the deformation of initially minor cracks since the onset of the flood season. The cracks are mainly attributed to the effects of heavy rainfall, which reduces slope stability. Additionally, the steep slope generates gravitational forces that induce downward soil movement and deformation, leading to crack formation. The observed cracks have the potential to expand and intensify, indicating ongoing deformation of the landslide.

### Variation of landslide monitoring curve

**Figure 6:**
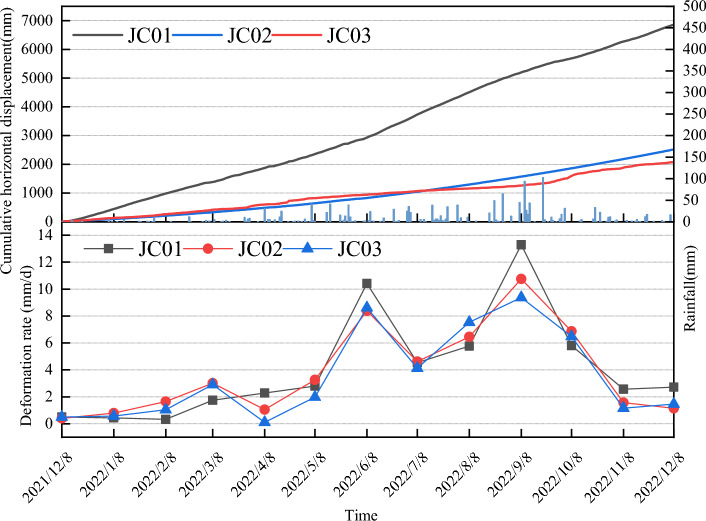
Cumulative surface horizontal displacement monitoring curves of the Piansongshu landslide.

The variation in landslide monitoring curves reflects the relationship between rainfall and displacement. Analyzing GNSS monitoring data from a section of the pine tree landslide, coupled with an assessment of regional rainfall patterns, reveals distinct behaviors during periods of concentrated and sparse rainfall (Fig. 6). The list of the monitoring data is given in Supplementary data. During concentrated rainfall, characterized by high intensity and a cumulative rainfall of 1055 mm, which constitutes a significant portion of the annual rainfall, the average daily rainfall is 43 mm, peaking at 102 mm in a single day. In contrast, sparse rainfall periods register lower intensities, with a cumulative rainfall of 80.4 mm. In the concentrated rainfall period, cumulative displacements at JC01, JC02, and JC03 reached 5186.84 mm, 1566.58 mm, and 1258.64 mm, respectively. Notably, JC01 exhibited greater cumulative horizontal displacements compared to JC02 and JC03. The slopes of the displacement–time curves displayed clear increases during this period, indicating significant deformation primarily influenced by the self-weight of the slope body. As rainfall diminishes, the slope's deformation rate decreases accordingly.

For the middle soil landslide, deformation is predominantly influenced by cumulative rainfall, with short-term heavy rainfall having a less pronounced effect on slope deformation, as evidenced by displacement–time curves. There exists a close correlation between deformation rate and rainfall in the study area. Monitoring points JC01, JC02, and JC03 exhibit average displacement and deformation rates of approximately 4.1 mm/day, 3.8 mm/day, and 3.5 mm/day, respectively. Deformation rates peak during concentrated rainfall periods and remain lower during sparse rainfall periods.

The cyclic nature of displacement growth rates in rainfall-type landslides aligns with the rainy season, with no continuous increase over time. During sparse rainfall stages, displacement growth rates are minimal or non-existent, fluctuating as rainfall increases, and escalating significantly during periods of sudden rainfall influx. Heavy rainfall emerges as a crucial trigger for biased pine landslide deformation, underscoring the importance of enhanced slope body monitoring during the rainy season.

## Methods

### The transfer coefficient method

(1) Stability coefficient calculation formula

1 $$F_{s} = \frac{{\sum\limits_{i = 1}^{n - 1} {\left( {R_{i} \prod\limits_{j = 1}^{n - 1} {\psi_{j} } } \right) + R_{n} } }}{{\sum\limits_{i = 1}^{n - 1} {\left( {T{}_{i}\prod\limits_{j = 1}^{n - 1} {\psi_{j} } } \right) + T_{n} } }}$$

2 $$R_{i} = \left[ {W_{i} \cos \alpha_{i} - Q_{i} \sin \alpha_{i} + D_{i} \sin \left( {\beta_{i} - \alpha_{i} } \right)} \right]\tan \varphi_{i} + c_{i} l_{i}$$

3 $$T_{i} = W_{i} \sin \alpha_{i} + Q_{i} \cos \alpha_{i} + D_{i} \cos \left( {\beta_{i} - \alpha_{i} } \right)$$

4 $$\psi_{j} = \cos \left( {\alpha_{i} + \alpha_{i + 1} } \right) - \sin \left( {\alpha_{i} { - }\alpha_{i + 1} } \right)\tan \varphi_{i + 1}$$

5 $$\prod\limits_{j = 1}^{n - 1} {\psi_{j} = } \psi_{i} \times\psi_{i + 1} \times\psi_{i + 2} \ldots \times\psi_{n - 1}$$

where R_i_ is the anti-sliding, D_i_ is the flow pressure, Q_i_ is the seismic force, ψ_i_ is the coefficient of transmission (j = i), W_i_ is the deadweight standard value, c_i_ is the cohesion standard value, φ_i_ is the standard value of internal friction angle, *D* is the flow pressure, α is the inclination angle of slip surface, β is the average dip angle of the groundwater flow line.

(2) Calculation formula of the residual sliding force of the landslide

6 $$P_{i} = P_{i - 1} \times\psi_{i - 1} + F_{st}\times T_{i} - R_{i}$$

where T_i_ is the anti-sliding force, P_i_ P_i-1_ is the residual downward slippery force, and F_st_ is the safety factor of the residual sliding force of the slider.

### The basic theory of rainfall infiltration

To gain a comprehensive understanding of the geological context in the study area, a field geological investigation of the landslide was conducted. Furthermore, the mechanism of rainfall-induced landslide deformation was examined using the GeoStudio software. Specifically, the SEEP/W software from the GeoStudio suite was employed to simulate and analyze the infiltration line, which represents variations in groundwater levels. Numerical simulation techniques were employed to determine the changes in pore water pressure within the slope by analyzing the infiltration line. The analysis utilized the seepage equation implemented in the SEEP/W module^27^.

7 $$\frac{\partial }{\partial x}\left( {k_{x} \frac{\partial h}{{\partial x}}} \right) + \frac{\partial }{\partial y}\left( {k_{y} \frac{\partial h}{{\partial y}}} \right) + q = \frac{\partial Q}{{\partial t}}$$

where h is the total head, k_x_ is the permeability coefficient in the x-direction, k_y_ is the permeability coefficient in the y-direction, q is the boundary recharge rate, Q is the water storage per unit volume, and t is the time.

When compared to the prediction accuracy for fine-textured soils, the prediction performance is favorable for coarse-grained soils, such as sand and sandy clay loam. Van-Genuchten (VG) introduced a mathematical model for fitting the soil–water characteristic curve. The model can be expressed as follows^28^:

8 $$\Theta = S_{e} = \left[ {\frac{1}{{1 + \left( {{\text{a}}\uppsi } \right)^{n} }}} \right]^{m}$$

The parameter relationships among a, n, and m significantly impact the prediction effectiveness of the VG model. The inclusion of the maximum matrix suction point is dependent on the dispersion and interval range of a, m, and n. The VG model aims to select parameter relationships that optimize the curve fitting degree, enhancing the accuracy of predictions.

### Numerical simulation

**Figure 7:**
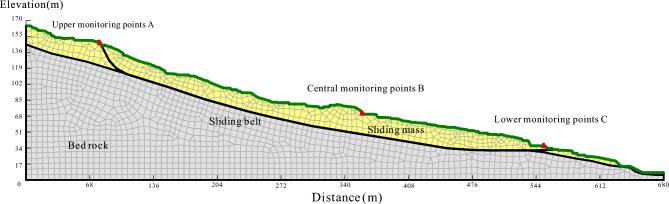
Calculation Model for the Sect. 2–2 of the Piansongshu Landslide.

To analyze the strong deformation observed in the 2–2′ section, a two-dimensional model was created using the finite element software GeoStudio. The model employed an Elastic–Plastic constitutive model and the Mohr–Coulomb strength criterion. The model consisted of three layers: an upper layer of landslide accumulation soil, a lower layer of slip zone soil, and an impermeable bedrock at the bottom (Fig. 7). The grid division pattern was chosen as quadrilateral and triangle, with 1984 nodes and 1847 cells.

To study the seepage field changes within the slope, three monitoring points were placed at different locations. During rainfall infiltration, the change of pore water pressure below the waterline was not significant. Therefore, three monitoring points were placed strategically above the waterline and near the surface of the slope to capture the impact of rainfall on the soil closer to the surface. The study aimed to explore the changes in pore water pressure and stability coefficient under different rainfall intensities, rainfall patterns, and long-term continuous rainfall conditions.

Based on the geotechnical physics and mechanics experiments and test data of similar landslides, the physical and mechanical parameters of each soil layer of the Piansongshu landslide were obtained (Table 1). The initial groundwater level was established from borehole data, determining the depth of the water table. In setting the parameters for the landslide simulation, the surface of the landslide was treated as a flow boundary, calibrated to the scenario where the rainfall intensity does not exceed the topsoil's permeability. This intensity was gauged using a 24-h maximum precipitation metric and historical rainfall records from the region, thereby incorporating the frequency and intensity of rainfall into the model. The landslide-prone area is frequently subjected to intense rainfall, with the maximum recorded daily rainfall reaching 243.3 mm. Consequently, the analysis constructed four distinct scenarios based on rainfall intensities: moderate rainfall at 45 mm/d, heavy rainfall at 90 mm/d, severe rainfall at 150 mm/d, and extreme rainfall at 243 mm/d.

**Table 1:** Physical and mechanical parameters of the Piansongshu landslide soil.

Soil type	Cohesion (kPa)	Internal friction angle (°)	Permeability coefficient (m/d)	Moisture content by volume	Elastic modulus (MPa)	Poisson ratio
Sliding-body	11.5	16.4	0.5	0.51	24	0.334
Sliding zone soil	14.6	13.3	1.00E–05	0.49	22	0.3
Sliding bed (bedrock)	0.22	35.01	–	0.05	2000	0.33

The strength parameters of the sliding and the sliding zone are the results under saturated conditions, and the remaining mechanical parameters are the average value of the sample.

The soil water characteristic curve was approximated using the Van Genuchten (VG) prediction model within the SEEP/W module, enabling the determination of the hydraulic conductivity function. The underlying bedrock was modeled as an impermeable boundary due to its significantly lower permeability coefficient when compared to the upper soil's saturated permeability coefficient.

## Results and discussion

### Stability calculation of transfer coefficient method

Among the three longitudinal profiles of the landslide, the stability coefficients indicate that the secondary shear along the 2–2′ profile, as well as the overall landslide calculation, are relatively lower compared to those along the 1–1′ and 3–3′ profiles. Consequently, the stability of the landslide is weaker in the middle section compared to the two sides (Table 2). These findings align closely with field investigations of landslide deformation and stability signs. The stability assessment reveals consistent results with the observed signs of landslide deformation in the field. The stability of potential secondary shear surfaces across the three profiles exhibits significant fluctuations. Under working condition 2, these surfaces fluctuate between unstable and marginally stable states. However, under working condition 1, the entire landslide is deemed stable. Specifically, the 2–2′ and 3–3′ profiles demonstrate basic stability under working condition 2, whereas the 1–1′ profile remains in a stable condition.

**Table 2:** Landslide stability coefficient calculation results.

Section		Condition	safety factor	stability factor	steady state
1–1′	The secondary shear surface	Condition 1	1.1	1.126	Basically stable
		Condition 2	1.1	1.029	Not stable
	The primary sliding surface	Condition 1	1.16	1.275	Stability
		Condition 2	1.16	1.151	Stability
2–2′	The secondary shear surface	Condition 1	1.1	1.11	Basically stable
		Condition 2	1.1	1.015	Not stable
	The primary sliding surface	Condition 1	1.16	1.236	Stability
		Condition 2	1.16	1.146	Basically stable
3–3′	The secondary shear surface	Condition 1	1.1	1.097	Basically stable
		Condition 2	1.1	1.025	Not stable
	The primary sliding surface	Condition 1	1.16	1.252	Stability
		Condition 2	1.16	1.144	Basically stable

Condition 1 (natural condition), Condition 2 (rainstorm conditions).

### Variation of the seepage field

**Figure 8:**
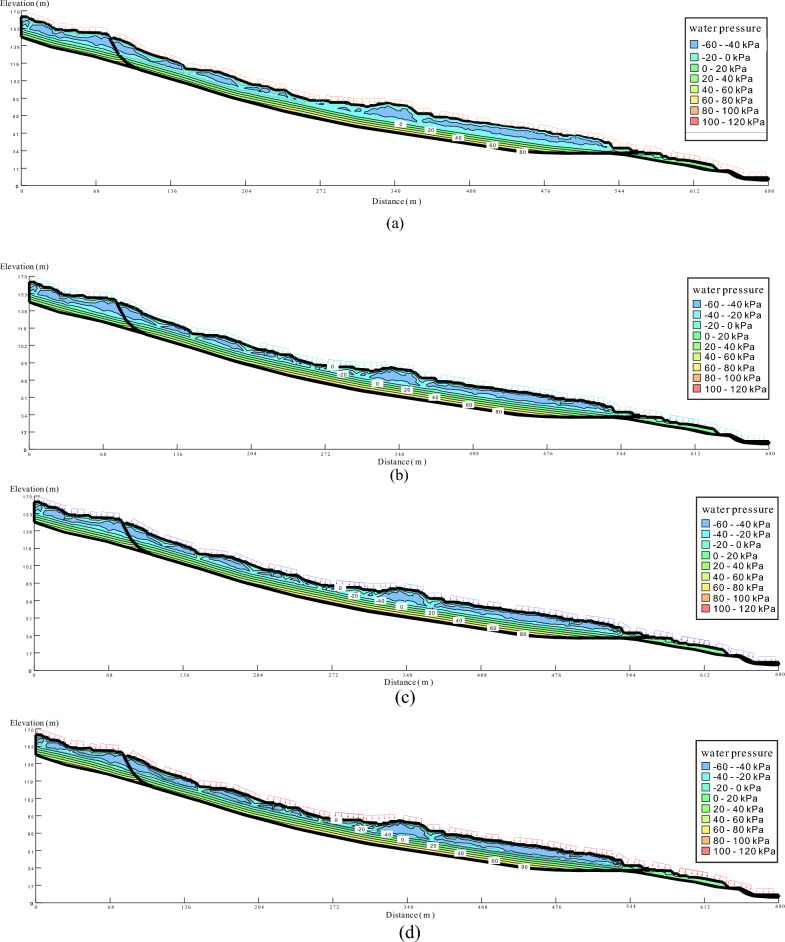
Water pressure distribution in the Piansongshu landslide under different rainfall conditions.

**Figure 9:**
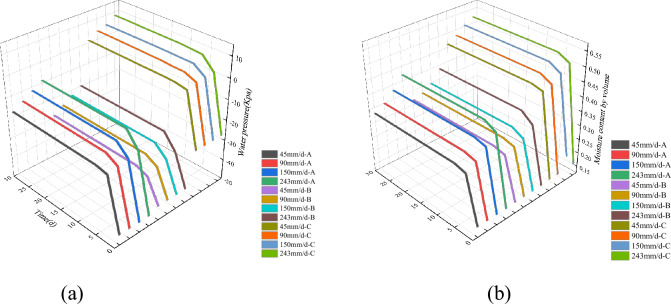
Variations in Pore Water Pressure and Volumetric Water Content at Different Monitoring Points. (**a**) Changes in Water Pressure; (**b**) Changes in Volumetric Water Content.

With an increasing intensity of rainfall, the water pressure on the slope exhibits a continuous rise, indicating a transition from unsaturated to saturated conditions (Fig. 8). At monitoring point, A, the pore water pressure undergoes a rapid increase during the initial six days of rainfall. The rate of change in pore water pressure intensifies with the increasing intensity of rainfall. Under intense rainfall conditions with an intensity of 243 mm/d, the volumetric moisture content rises from 0.16 to 0.42 (Fig. 9). The rate of change in volumetric moisture content in the upper part of the slope exhibits significant variation as rainfall intensity increases. Relative to the initial state, the volumetric moisture content of the slope continues to escalate. As the rainfall intensity increases, the volumetric moisture content that saturates the slope also increases, resulting in a shorter time for the slope to reach saturation. The fluctuation of water pressure intensifies as the intensity of rainfall increases. During periods of heavy rainfall, the time required for water pressure to rise and stabilize decreases. Additionally, the matrix suction of the slope diminishes, and localized shallow saturation zones may emerge. In contrast to the upper monitoring points, the rate of change of volumetric water content at central monitoring point B exhibits a decrease. Moreover, the rate of change of volumetric water content diminishes further after the rainfall has persisted for 30 days in comparison to the previous stages of rainfall.

The volumetric moisture content of the lower part of the slope is generally higher than that of the upper part. Consequently, rainwater accumulates at the base of the slope, leading to a relatively rapid saturation of the slope. At monitoring point C, the volumetric moisture content of the slope increases from 0.17 to 0.46 after a 30-day period with a rainfall intensity of 45 mm/d. As the rainfall intensity increases to 90 mm/d, the volumetric water content inside the slope continues to rise, reaching a value of 0.48 from an initial value of 0.17. Under a rainfall intensity of 150 mm/d, the water pressure increases from 0.17 to 0.50. Furthermore, under the condition of heavy rainfall with an intensity of 243 mm/d, the water pressure rises from 0.17 to 0.52.

In general, the upper monitoring point A demonstrates the most pronounced fluctuations in water pressure. With the increasing intensity of rainfall, the instantaneous rainfall intensity also gradually rises, leading to a higher rate of change in pore water pressure during the early stages of rainfall. During the initial phase of rainfall, the pore water pressure in the upper section of the slope exhibits a more rapid increase compared to the lower section. However, during the later stage of rainfall, the shallow soil in the slope, being closer to saturation, experiences limited infiltration capacity, resulting in the cessation of pore water pressure increase in the shallow soil region.

As rainfall intensity escalates, so does the rate of volumetric moisture content change, leading to more marked deformation in the landscape. With consistent rainfall duration, the internal slope’s volume water content rises more swiftly with higher rainfall intensities. Nonetheless, this effect is confined to the uppermost layers of the soil, a few meters down, and becomes significant only during prolonged rainfall events. With equal duration of rainfall, higher intensities lead to increased cumulative precipitation and deeper infiltration. Once the cumulative rainfall surpasses a threshold, the probability of landslide occurrence spikes, particularly under conditions of intense rainfall.

### Change of saturation line

**Figure 10:**
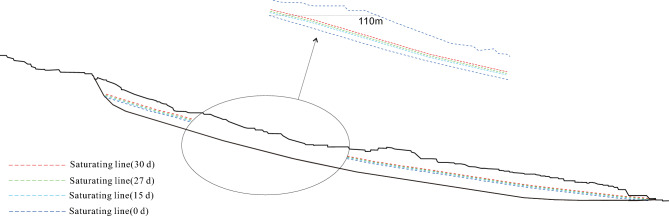
Variations of the Saturation Line at the Upper Position of the Slope.

As rainfall intensity increases, the substrate suction decreases, and the infiltration line steadily rises. This process results in the expansion of the slope saturation zone, as indicated by the increasing infiltration line and the extent of the saturation zone over time (Fig. 10). Throughout the 30-day rainfall period, the infiltration line consistently moved upwards. After the rainfall, the infiltration line near the foot of the slope reached the crest of the slope. However, due to the elongated nature of the slope, the infiltration line at deeper locations within the slope remained at a lower elevation. Consequently, after the rainfall event, two distinct infiltration lines were observed within the slope, with one positioned near the surface and the other located at a greater depth within the slope.

Under intense rainfall conditions, the near-surface soil of the slope experiences temporary saturation, resulting in variations in pore water pressure that propagate from the surface towards the interior of the slope. Initially, there is an increase in pore water pressure, followed by a gradual decrease over time.

### Stability evaluation

**Figure 11:**
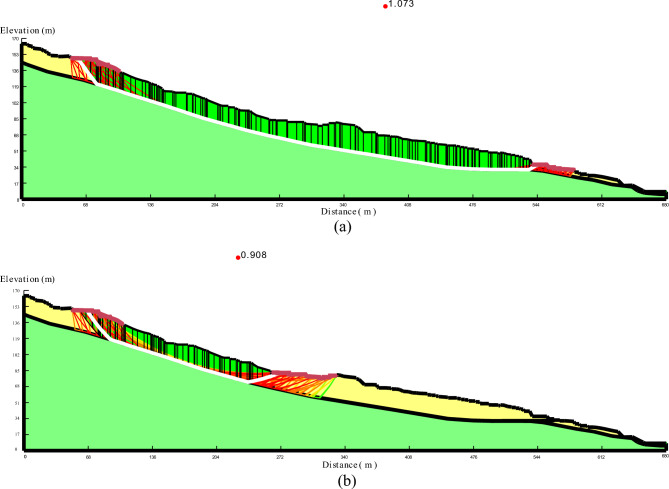
Critical sliding surface of the landslide.

**Figure 12:**
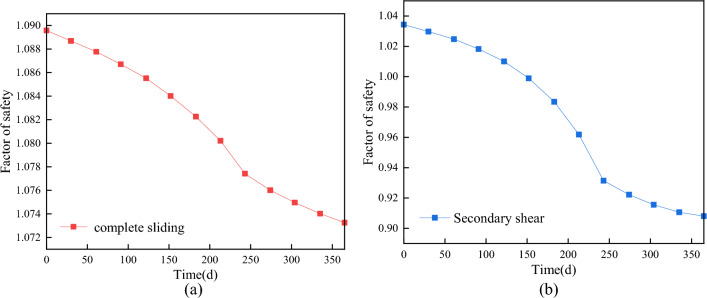
Stability coefficient variations over time. (**a**) Complete sliding; (**b**) Secondary shear.

As the duration of rainfall increases, the magnitude of the decrease in the stability coefficient becomes more pronounced. The infiltration of rainwater into the slope results in an increase in soil moisture content, an expansion of the saturation range, a reduction in matrix suction and shear strength, and a decrease in the stability coefficient of the slope, especially with higher rainfall intensities. This phenomenon leads to a general reduction in the stability of the slope, as depicted in Fig. 11. When comparing the stability coefficients of the overall sliding surface and the secondary shear surface of the landslide, it is observed that the stability coefficient of the secondary shear surface experiences more pronounced changes than that of the overall sliding surface, as illustrated in Fig. 12. Moreover, the stability coefficient exhibits a greater decrease with longer durations of rainfall.

The safety coefficient of slope stability is influenced by different types of rainfall, even under the same total rainfall conditions, as depicted in Fig. 11. Generally, as rainfall infiltrates into the slope, the safety coefficient gradually decreases. Among the different types of rainfall, decreasing type rainfall exhibits the greatest decrease in the stability coefficient, followed by increasing type rainfall and centralized type rainfall. Average rainfall causes a slightly smaller decrease in the stability coefficient. The stability coefficient experiences the most significant decrease during the early period of decreasing rainfall, while the center rainfall shows the most pronounced decrease during the middle period of rainfall. The rate of incremental rainfall increase reaches its maximum during the late rainfall period. The longer the rainfall duration, the greater the decrease in the stability coefficient, and the longer the time required for the stability coefficient to return to a stable state after the rainfall stops. As the rainwater dissipates and the water content in the soil volume decreases, the matrix suction increases, leading to an increase in the stability coefficient of the slope until it reaches a stable state.

**Figure 13:**
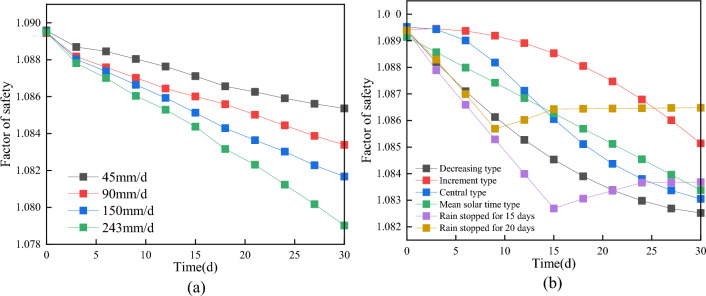
The change of landslide stability coefficient. (**a**) Effect of rainfall intensity on stability coefficient; (**b**) Effect of rain pattern on stability coefficient.

For the overall sliding surface of the landslide, the slope's stability coefficient consistently decreases as the rainfall intensity increases. The rate of decrease is more pronounced during the early stage of rainfall (0 days-3 days), becomes slower during the middle stage (3 days–15 days), and then accelerates again after 15 days of rainfall. In the final stage of rainfall, the slope is on the verge of destruction, and the stability coefficient decreases significantly faster under a rainfall intensity of 243 mm/d compared to lower intensities (Fig. 13).

During the initial stage of rainfall, the soil exhibits lower water content. As rainwater infiltrates into the soil, the moisture content within the slope rapidly increases, leading to a decrease in matrix suction and subsequent reduction in shear strength. This causes the stability coefficient to decrease at a higher rate. Once the water content in the upper part of the slope reaches a certain threshold, it cannot accommodate further infiltration of a large amount of rainfall. Consequently, the permeability coefficient of the surface soil of the slope decreases, causing rainfall to converge and form surface runoff that drains away. At this point, the safety coefficient of the slope no longer experiences a significant decrease.

### Deformation mechanism

Under the rainfall, rainwater infiltrates along the slope surface, so that the permeability within the slide gradually spreads outward, while the buoyancy support of the landslide becomes smaller, thus affecting the stability of the landslide. The influence of rainfall infiltration on the seepage field is mainly concentrated in the surface layer of the slope, with the increase of rainfall holding time, the surface layer of the slope soil from unsaturated to saturated, the surface layer of the slope forms a transient saturated zone, the rainwater is constantly infiltrated by the surface layer of the soil to the soil deeper, the longer the duration of rainfall, the larger the rainfall infiltration, the smaller the unsaturated zone in the soil. With the enhancement of rainfall, the displacement of the landslide surface becomes larger and larger in extent. When the intensity of rainfall increases, the decreasing trend of the coefficient of safety also increases, and the pore water pressure and matrix suction of the landslide are much lower, resulting in a decrease in the shear strength of the geotechnical soil, which in turn results in a rapid decrease in the coefficient of stability.

## Conclusions

The spatial distribution pattern of landslides in Wanzhou District exhibits a strong correlation with geological formations, topography, and water distribution. The predominant type of landslides in the region is accumulation landslides.

The Piansongshu landslide was developed in the Middle Triassic Badong Formation, a slide-prone stratum, and rainfall is the main inducing factor for landslide deformation. The determination of the landslide boundary is mainly based on the deformation. The trailing edge of the landslide is mainly bounded by the actual existing fractures, with secondary shear zones in the middle of the landslide. According to the field geological survey results, the landslide deformation is strong in the middle and gradually weakening on both sides, and the stability calculation results are consistent with the signs of landslide deformation in the field survey.

As rainfall persists, rainwater infiltrates from the surface layer of the slope to deeper regions. The duration of rainfall directly impacts the extent of rainwater infiltration, with longer durations resulting in greater infiltration. Consequently, the soil on the slope surface transitions from an unsaturated to a saturated state, leading to a reduction in the unsaturated area within the slope. At the front edge of the mound, the saturation line assumes a linear form due to higher permeability and shorter seepage paths, extending towards the surface in regions experiencing greater deformation. Over time, as rainfall intensity diminishes during the non-rainy season, the saturation line recedes.

The stability coefficient of the secondary shear surface of the landslide exhibits greater variability compared to the overall sliding surface. As the duration of rainfall increases, the stability coefficient experiences a more significant decrease. In general, the slope safety coefficient gradually decreases over time under different rainfall conditions, attributable to the infiltration of rainfall. Even after the rain ceases, the stability coefficient continues to decrease. However, over time since the rain stopped, the slope stability coefficient gradually increases until it reaches a stable state.

## Supplementary Information

Supplementary Information.

## Data Availability

The datasets generated and analyzed during the current study are not publicly available due to the funding responsibility but are available from the corresponding author on reasonable request.

## References

[1] Tang, H. M., Wasowski, J. & Juang, C. H. Geohazards in the three Gorges Reservoir Area, China—Lessons learned from decades of research. *Eng. Geol.* **261**, 105267 (2019).

[2] Iqbal, J., Dai, F. C., Hong, M., Tu, X. B. & Xie, Q. Z. Failure mechanism and stability analysis of an active landslide in the Xiangjiaba Reservoir Area, Southwest China. *J. Earth Sci.* **29**, 646–661 (2018).

[3] Chen, Y. *et al.* Review of landslide susceptibility assessment based on knowledge mapping. *Stoch. Environ. Res. Risk Assess.* **36**, 2399–2417 (2022).

[4] Wu, R. A. *et al.* Landslide susceptibility assessment in mountainous area: A case study of Sichuan-Tibet railway, China. *Environ. Earth Sci.* **79**, 1–16 (2020).

[5] Xue, Y., Miao, F. S., Wu, Y. P., Li, L. W. & Meng, J. J. Application of uncertain models of sliding zone on stability analysis for reservoir landslide considering the uncertainty of shear strength parameters. *Eng. Comput.* **38**, 3057–3076 (2022).

[6] Zhang, Y. G., Zhu, S. Y., Tan, J. K., Li, L. D. & Yin, X. J. The influence of water level fluctuation on the stability of landslide in the Three Gorges Reservoir. *Arab. J. Geosci.* **13**, 1–10 (2020).

[7] Wu, Y., Huang, S., Liu, K., Zhang, Q. & Pan, H. Study on physical and mechanical characteristics of shear band in Jinpingzi Landslide Region II. *Front. Phys.* **10**, 857274 (2022).

[8] Summa, V., Sinisi, R., Paris, E. & Bonomo, A. E. Compositional features of fine sediments involved in the montescaglioso landslide (Southern Italy). *J. Earth Sci.* **33**, 1513–1525 (2022).

[9] Chinkulkijniwat, A. *et al.* Stability characteristics of shallow landslide triggered by rainfall. *J. Mt. Sci.* **16**, 2171–2183 (2019).

[10] Du, G. L., Zhang, Y. S., Yao, X., Yang, Z. H. & Yuan, Y. Field investigations and numerical modeling of a giant landslide in the region of Eastern Himalayan Syntaxis: Jiaobunong landslide. *J. Mt. Sci.* **18**, 3230–3246 (2021).

[11] Beyene, A., Tesema, N., Fufa, F. & Tsige, D. Geophysical and numerical stability analysis of landslide incident. *Heliyon.* **9**, e13852 (2023). 36873491 10.1016/j.heliyon.2023.e13852 PMC9982034

[12] Miao, F. S., Wu, Y. P., Ákos, T., Li, L. W. & Xue, Y. Centrifugal model test on a riverine landslide in the Three Gorges Reservoir induced by rainfall and water level fluctuation. *Geosci. Front.* **13**, 101378 (2022).

[13] Huang, F. M. *et al.* Uncertainty pattern in landslide susceptibility prediction modelling: Effects of different landslide boundaries and spatial shape expressions. *Geosci. Front.* **13**, 101317 (2022).

[14] Yang, H. *et al.* Permeability of the reservoir water fluctuation zone of landslide bodies in the Three Gorges Reservoir Area, China. *Arab. J. Geosci.* **13**, 1–14 (2020).

[15] Luo, G. L., Ren, G. M., Bao, X. J., Yang, X. L. & Liu, T. Stability analysis of the shiliushubao landslide based on deformation characteristics and external trigger factors in the Three Gorges Reservoir. *Adv. Civil Eng.* **2021**, 1–12 (2021).

[16] Zhang, Z. L. & Wang, T. Stability and deformation of Xiaozhuang landslide: A large-scale creeping landslide in Gansu, China. *J. Mt. Sci.* **19**, 756–770 (2022).

[17] Naidu, S., Sajinkumar, K. S., Thomas Oommen, V. J. A., Samuel, R. & Muraleedharan, C. Early warning system for shallow landslides using rainfall threshold and slope stability analysis. *Geosci. Front.* **9**, 1871–1882 (2018).

[18] Wang, L. Q. *et al.* Stability analysis of the Xinlu Village landslide (Chongqing, China) and the influence of rainfall. *Landslides* **16**, 1993–2004 (2019).

[19] Kaya, A. & Midilliü, M. Slope stability evaluation and monitoring of a landslide: A case study from NE Turkey. *J. Mt. Sci.* **17**, 2624–2635 (2020).

[20] Chen, H. Y. *et al.* Inducing factors and deformation mechanism of the Zhangjiacitang landslide in the Three Gorges Reservoir Area. *Sci. Rep.* **13**(1), 12926 (2023). 37558819 10.1038/s41598-023-40186-6 PMC10412534

[21] Gong, Q. H., Wang, J., Zhou, P. & Guo, M. A. Regional landslide stability analysis method under the combined impact of rainfall and vegetation roots in South China. *Adv. Civil Eng.* **2021**, 1–12 (2021).

[22] Zhang, Y. G. *et al.* Prediction of landslide displacement with dynamic features using intelligent approaches. *Int. J. Min. Sci. Technol.* **32**, 539–549 (2022).

[23] Xu, J. & Zhao, Y. N. Stability analysis of geotechnical landslide based on GA-BP neural network model. *Comput. Math. Methods Med.* **2022**, 1–10 (2022). 10.1155/2022/3958985 PMC9236796 35770123

[24] Cheng, J. *et al.* Landslide susceptibility assessment model construction using typical machine learning for the Three Gorges Reservoir Area in China. *Remote Sens.* **14**, 2257 (2022).

[25] Saha, S., Saha, A., Hembram, T. K., Pradhan, B. & Alamri, A. M. Evaluating the performance of individual and novel ensemble of machine learning and statistical models for landslide susceptibility assessment at Rudraprayag District of Garhwal Himalaya. *Appl. Sci.* **10**, 3772 (2020).

[26] Pourghasemi, H. R., Sadhasivam, N., Amiri, M., Eskandari, S. & Santosh, M. Landslide susceptibility assessment and mapping using state-of-the art machine learning techniques. *Nat. Hazards.* **108**, 1291–1316 (2021).

[27] Ma, S. *et al.* Topographic changes, surface deformation and movement process before, during and after a rotational landslide. *Remote Sens.* **15**, 662 (2023).

[28] Tong, J. *et al.* Study on synergistic characteristics of accumulation landslides supported by arbor species. *Forests* **13**, 1610 (2022).

